# Central Portalization Correlates with Fibrosis but Not with Risk Factors for Nonalcoholic Steatohepatitis in Steatotic Chronic Hepatitis C

**DOI:** 10.1155/2014/329297

**Published:** 2014-11-30

**Authors:** Hwajeong Lee, Sanaz Ainechi, Karen Dresser, Elizabeth M. Kurian

**Affiliations:** ^1^Anatomic Pathology, Albany Medical College, 47 New Scotland Avenue, MC81, Albany, NY 12208, USA; ^2^Anatomic Pathology, University of Massachusetts, 1 Innovation Drive, Biotech 3, Worcester, MA 01605, USA; ^3^Anatomic Pathology, University of Texas Southwestern Medical Center, 5323 Harry Hines Boulevard, Dallas, TX 75390-9234, USA

## Abstract

Concomitant steatosis in chronic hepatitis C is associated with fibrosis and unfavorable treatment outcome. Central zone injury in nonalcoholic steatohepatitis (NASH) manifests as central portalization, with centrizonal microvessels and ductular reaction. We investigated whether central portalization in steatotic HCV biopsies would identify patients with metabolic risk factors for NASH. Liver biopsies with chronic hepatitis C and >10% steatosis (*n* = 65) were evaluated for the degree of steatosis, zonation of steatosis, fibrosis, and nonalcoholic fatty liver disease (NAFLD) activity score. The presence of centrizonal microvessels, sinusoidal capillarization, ductular reaction, and CK7 positive intermediate-phenotype hepatocytes were evaluated by CD34 and CK7 immunostain. The degree of steatosis and fibrosis showed a positive correlation. Additional positive correlations were noted between centrizonal angiogenesis and NAFLD activity score and central portalization and fibrosis. However, neither central portalization nor zonation of steatosis identified patients with metabolic risk factors for NASH. Therefore, central portalization cannot be used as a surrogate marker to identify patients with metabolic risk factors for NASH in steatotic HCV biopsies. The mechanism of centrizonal injury in steatotic HCV hepatitis is not solely attributable to the metabolic risk factors for NASH.

## 1. Introduction

In chronic hepatitis C, steatosis is a common histologic finding. Studies have categorized the steatosis of chronic HCV into viral type versus metabolic type. The “viral” type of steatosis is well demonstrated in HCV genotype 3; these liver biopsies show a greater amount of steatosis compared to those of non-3 genotypes [1–4]. Experimental studies suggest that the HCV viral core protein impairs lipid oxidation and induces accumulation of triglyceride in the hepatocytes [5–7]. Furthermore, the degree of steatosis is related to the viral load [4–6, 8, 9], and the amount of steatosis decreases following treatment [10–12]. In the setting of liver transplantation, the hepatic steatosis is an early indicator of HCV reinfection [13]. The second type of “metabolic” steatosis is usually demonstrated in non-3 genotypes and is associated with risk factors of nonalcoholic fatty liver disease, such as high body mass index (BMI) [1, 4, 14–16]. In these patients, insulin resistance enhances peripheral lipolysis and hepatic lipogenesis [17], while impairing the export of triglycerides from the hepatocytes [6]. Thus, insulin resistance appears to play a key role in the development of metabolic steatosis [14]. Since chronic hepatitis C patients are also at risk for impaired glucose metabolism and demonstrate a higher homeostatic model assessment (HOMA-IR; a surrogate marker of insulin resistance), viral and metabolic factors are not mutually exclusive in the pathogenesis of steatosis [6, 18–21].

Retrospective and longitudinal studies show that the degree of steatosis in chronic hepatitis C is positively correlated with the degree of fibrosis [1, 4, 6, 16, 22, 23], and steatosis/fibrosis negatively affects the likelihood of achieving a sustained virologic response following treatment [6, 10, 15, 23–26]. Similarly, hepatitis C biopsies with concomitant NASH were more frequently associated with advanced fibrosis compared to those without NASH [2, 7, 15]. Moreover, superimposed hepatic steatosis in chronic hepatitis C appears to pose an increased risk of hepatocellular carcinoma [27, 28]. The current literature suggests that management of metabolic risk factors for NASH, such as obesity and type 2 diabetes mellitus (DM), may alter the disease progression in chronic hepatitis C [3, 21, 29–33].

A recent study showed that biopsies of NASH exhibit increased vascular channels and ductular reaction in central zones, termed central portalization, and it was more common in advanced fibrosis [34]. We investigated whether the central portalization in steatotic HCV biopsies would identify a subset of patients with metabolic risk factors for NASH. If the groups with or without risk factors for NASH demonstrate a difference in the degree of vascular proliferation and ductular reaction in the central zone, the histologic findings may be used as a surrogate marker to identify a subset of chronic hepatitis C patients at risk for superimposed NASH with its adverse outcome.

## 2. Materials and Methods

### 2.1. Study Group and Control Groups

The study was approved by the institutional review board. Liver biopsies from HCV hepatitis with concurrent macrovesicular steatosis (ranging from 10% to 80%), during the period of 2006–2008, constituted the study group (*n* = 65). Demographic and clinical data including the presence of type 2 diabetes mellitus (DM), obesity (body mass index (BMI) ≥ 30 kg/m^2^), hyperlipidemia, history of alcohol use, HCV genotypes, and treatment history were obtained from the electronic medical records.

For control groups, 22 cases of nonalcoholic steatohepatitis (NASH) biopsies with no history of HCV and 20 cases of HCV hepatitis biopsies with ≤10% of steatosis were retrieved. Relevant demographic and clinical data were obtained from the electronic medical records.

### 2.2. H&E and Trichrome Stain Review

Each liver biopsy was evaluated for the amount of steatosis and fibrosis. Ishak's fibrosis staging system was used for the study group and HCV control group [35]. Fibrosis staging system by NASH Clinical Research Network (CRN) was used for NASH control group [36]. The central zones around terminal hepatic veins were defined as previously published [37], and the number of central zones was recorded for each biopsy. For the study group, the zonation of steatosis (zone 3 or random) was evaluated. In addition, NAFLD activity score was calculated using the published criteria—degree of steatosis (5–33%: 1, >33–66%: 2, >66%: 3), ballooning (few: 1, many: 2), and lobular inflammation (<2 foci: 1, 2–4 foci: 2, >4 foci: 3; counted in 20x fields)—for the study group and NASH control group [36]. The presence of arterioles with muscular vessel wall in the central zone was evaluated on H&E stain.

### 2.3. Immunohistochemical Stain

The immunohistochemical stain for CK7 (1 : 200, Dako M7018, mouse monoclonal, clone OV-TL 12/20), CK19 (1 : 50, Dako M0888, mouse monoclonal, clone RCK108), and CD34 (1 : 160, Dako M7165, mouse monoclonal, clone QBEnd 10) was performed on the 5-micrometer thick tissue sections of paraffin-embedded tissue blocks. Antigen retrieval was carried out with 0.01 M citrate buffer at pH 6.0. The slides were stained on the Dako Autostainer (Dako Corporation, Carpinteria, CA).

Sections of various control tissues (breast for CK7, liver for CK19, and colon for CD34) with known positivity for the target proteins were used as positive stain controls. Positive staining was defined as dark brown staining pattern. Scant or fine granular background staining or no staining at all was considered negative.

### 2.4. Evaluation of Central Portalization

Microvessel and ductular reaction in the central zone was evaluated by CD34 and CK7, respectively. As described previously [38], microvessels are defined as small lumen-forming vascular channels with CD34 positive endothelial lining, without a visible muscular wall. Ductular reaction is defined as the presence of CK7 positive biliary-type cells arranged in ductular configuration [38]. Sinusoidal CD34 staining without complete lumen-forming channels was interpreted as sinusoidal capillarization. CK7 positive hepatocytes in the central zone, without morphologic ductule formation, were considered intermediate-phenotype hepatocytes [38]. Initially, the number of central zones with microvessels, sinusoidal capillarization, ductular reaction, and intermediate-type hepatocytes were evaluated, respectively. However, the distinction between microvessels and sinusoidal capillarization was difficult in some cases. Since the results of statistical analyses did not differ when sinusoidal capillarization was combined with microvessels, no distinction was made for the purpose of scoring, and sinusoidal capillarization was included in the count of microvessels (see below *M* score). For the same reasons, the intermediate-phenotype hepatocytes were included in the count of ductular reaction (see below *D* score).

Any zone with CK19 positivity was excluded from enumeration. The CK19 positive areas represent portal tracts or tangentially sectioned extensions of fibrous septa surrounding portal tracts, which may mimic central zones (see Section 4). The *M* score is defined as the number of central zones with microvessels and sinusoidal capillarization divided by the total number of central zones—the ratio of central zones with angiogenesis to the total number of central zones, within a biopsy. For example, in a biopsy with 10 central zones, if 2 central zones show microvessels and 3 central zones show sinusoidal capillarization, the *M* score is (2 + 3) divided by 10 = 0.5. The *D* score is defined as the number of central zones showing ductular reaction (including intermediate-type hepatocytes) divided by the total number of central zones in a biopsy. Cases displaying less than 4 central zones were considered inadequate for evaluation and were excluded from the statistical analysis (*n* = 18).

### 2.5. Statistical Analysis

Fisher's exact test was used when determining whether there are nonrandom associations between two categorical variables. Student's *t*-test was used when comparing the means of two groups. Pearson's correlation coefficient was used for correlation of two parameters. A *P* value of less than 0.05 was considered statistically significant.

## 3. Results

### 3.1. Study Group: Clinical Data and Review of Histology

Thirty-four (52%) patients had one or more risk factors, and 21 (32%) patients had no risk factors for NASH. The most common risk factor for NASH was obesity (Table 1). When only patients with one or more metabolic risk factors were considered, 97% (33/34) of them had obesity. The HCV genotype was available in 44 patients; all genotype 3 cases belonged to the study group (Table 1). Thirteen patients had been treated for HCV hepatitis prior to the biopsies. Clinical follow-up for twelve months or longer was available in 55 patients, with a mean follow-up period of 48 months (12–72 months). During the follow-up period, 6 (11%) subsequently developed clinical NASH or type 2 DM, and 16 (29%) experienced adverse outcomes, including relapse after treatment (*n* = 2), no response to treatment (*n* = 3), discontinuation of treatment due to side effects (*n* = 5), progression of fibrosis, hepatic decompensation, and development of hepatocellular carcinoma (*n* = 6).

On histologic review, the zonation of steatosis (zone 3 steatosis) was appreciated in 6 cases, of which 4 had metabolic risk factors for NASH. When the fibrosis, steatosis, and NAFLD activity score were compared between the subgroups with and without clinical parameters (categorical variables) among the study group, only the degree of steatosis between the obese and nonobese group showed a statistically significant difference (Table 2). Combination of the study group and HCV control group with ≤10% steatosis showed an even greater difference, with an average steatosis of 48% in the obesity group and 29% in the nonobese group (*P* = 0.001 by Student's *t*-test).

On average, 6 (range 1–22) central zones were identified per biopsy. The mean Ishak's fibrosis stage was 2.9 for the biopsies with adequate number of central zones (>3) and 4.3 for the biopsies with inadequate number (≤3) of central zones (*P* = 0.019 by Student's *t*-test). Similarly, the number of central zones showed an inverse relationship to fibrosis by Pearson's correlation test (Table 3). No statistically significant correlation was found between the fibrosis and steatosis in the study group. However, when the control HCV group with ≤10% of steatosis was combined with the steatotic HCV study group, there was a positive correlation between the degree of steatosis and Ishak's fibrosis stage (*r* = 0.26 by Pearson's correlation coefficient; *P* = 0.046).

Two cases showed arterioles in the central zone.

### 3.2. Study Group: Evaluation of Central Portalization

**Figure 1:**
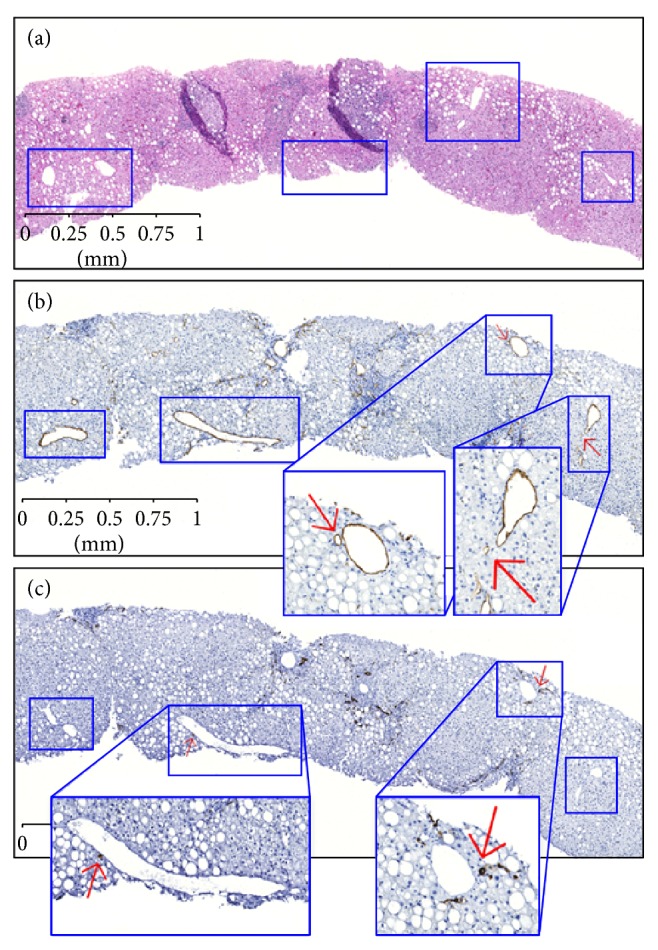
(a) Low magnification view of steatotic HCV liver biopsy with 4 central zones marked by squares (H&E, ×50). (b) Level section of the same liver biopsy demonstrates two central zones with microvessel and sinusoidal capillarization. In this case, the *M* score is 2/4 = 0.5. Insets: high magnification of microvessel/sinusoidal capillarization highlighted by CD34 immunostain and arrows (CD34, ×50; insets: CD34, ×200). (c) Level section of the same liver biopsy demonstrates two central zones with ductular reaction and intermediate-phenotype hepatocytes (CK7, ×50). In this case, the *D* score is 2/4 = 0.5. Insets: high magnification of ductular reaction/intermediate-phenotype hepatocytes highlighted by CK7 immunostain and arrows (CK7, ×50; insets: CK7, ×200).

A total of forty-seven cases were studied, after excluding cases with less than 4 central zones. All 47 (100%) cases showed microvessels and/or sinusoidal capillarization, with the average *M* score of 0.5 (range 0.1–1) (Figures 1(a) and 1(b)). Thirty-four (72%) of 47 cases showed ductular reaction and/or intermediate-phenotype hepatocytes, with the average *D* score of 0.3 (range 0-1) (Figures 1(a) and 1(c)). The *M* score showed a positive correlation with Ishak's fibrosis stage and NAFLD activity score. Similarly, the *D* score showed a positive correlation with fibrosis. Neither *M* nor *D* score showed statistically significant correlation with steatosis (Table 3).

No statistically significant difference was found in the *M* and *D* scores between the groups: with and without obesity, type 2 DM, hyperlipidemia, history of alcohol use, history of treatment, and HCV genotype 3. The result remained unchanged when the cutoff of steatosis was increased to 33% for the study group.

### 3.3. Control Groups

In the HCV control group, 15 cases were adequate for evaluation, and the *D* score showed a positive correlation with Ishak's fibrosis stage (Table 3). No statistically significant correlation was found between the *M* score and fibrosis, and the result remained unchanged when the cases with advanced fibrosis were only evaluated.

In the NASH control group without HCV, 64% of the biopsies showed low NASH CRN's fibrosis stage (0: 4 cases, 1a: 9 cases, 1b: 1 case, and 1c: 0 cases). No correlation was found between the *M* score and fibrosis, NAFLD activity score, and/or steatosis (Table 3). Ductular reaction was seen in only 3 of 15 cases; hence, statistical analysis was not performed.

## 4. Discussion

Chronic hepatitis C affects about 160 million worldwide [21] and is a significant cause of liver-related mortality [39, 40]. Hence, the staging liver biopsy for HCV hepatitis is a very common liver sample encountered in routine surgical pathologists' practice. Steatosis in HCV liver biopsies is common and is seen in approximately 74% of genotype 3 and 48% of nongenotype 3 biopsies [1]. Concomitant steatosis in chronic hepatitis C confers a worse prognosis in the progression of fibrosis, treatment outcome, and risk of hepatocellular carcinoma [1, 4, 6, 10, 15, 16, 22–33]. Moreover, nonalcoholic steatohepatitis (NASH) and its risk factors superimposed on chronic hepatitis C are associated with progression of fibrosis, suggesting that modification of the metabolic risk factors for NASH might be beneficial for these patients [2, 3, 15, 21].

The current study confirms that obesity is a dominating metabolic risk factor in steatotic HCV biopsies, and the biopsies from patients with obesity demonstrate a higher degree of steatosis than those without obesity. All genotype 3 biopsies demonstrated more than 10% of steatosis, supporting the role of a viral factor in steatosis.

Our results also confirm the previous findings that steatosis and fibrosis are positively correlated in chronic hepatitis C [1, 4, 6, 16, 22–25]. Furthermore, the study group contained more fibrosis than the HCV control group. However, when HCV biopsies with >10% steatosis are only considered, the degree of steatosis does not correlate with fibrosis.

All the study groups showed either microvessels or sinusoidal capillarization in the central zone, and the *M* score showed a positive correlation with the NAFLD score and fibrosis. This result suggests the role of central zone injury and angiogenesis in the progression of fibrogenesis in steatotic HCV hepatitis, similar to NASH [34]. The association between angiogenesis, steatosis, and chronic hepatitis C was addressed in prior studies [41–43]. Kukla et al. studied 35 steatotic liver biopsies of chronic hepatitis C in comparison to 37 nonsteatotic biopsies and showed that higher grade of steatosis was associated with advanced fibrosis and angiogenesis [41]. Their study excluded genotype 3 and patients with risk factors for NASH, and the central zone was not separately evaluated. However, the authors observed higher CD34 expression in the “lobules” and “fibrous septa in the lobules” of the biopsies with >66% of steatosis. Thus, the result seems to suggest a positive correlation between steatosis and centrizonal angiogenesis in steatotic HCV biopsies in the absence of risk factors for NASH [41].

There are two possible mechanisms for angiogenesis in chronic liver diseases. Preexisting vasculature may grow and branch as a part of tissue repair in response to inflammation by recruitment of proangiogenic markers. This process is visualized as predominantly periportal vasculature with inflammatory activity and fibrosis in chronic hepatitis C biopsies [42]. In contrast, in conditions of centrizonal ischemia, angiogenesis may be induced by tissue hypoxia, via intrahepatic vascular remodeling and sinusoidal capillarization, and upregulation of proangiogenesis in extracellular matrix-producing cells, including hepatic stellate cells [43–48]. This ischemia-driven centrizonal angiogenesis has been postulated in the pathogenesis of central portalization in NASH [34]. The positive correlation between the *M* score and NAFLD activity score and the fact that leptin, a circulating peptide hormone mainly produced by adipose tissue, leads to upregulation of proinflammatory and proangiogenic cytokines in human hepatic stellate cells [45] raise a possibility that the latter mechanism may be involved in the angiogenesis of steatotic chronic hepatitis C. Future correlation studies between the distribution of the hepatic stellate cells and angiogenesis in steatotic chronic hepatitis C may be insightful.

Centrizonal ductular reaction and intermediate-phenotype hepatocytes seem to correlate with fibrosis in chronic hepatitis C biopsies, regardless of steatosis. This is shown by the positive correlation between the *D* score and fibrosis in the study group, as well as the HCV control group. Centrizonal ductular reaction is speculated to represent an end-product of metaplasia or dedifferentiation of parenchymal hepatocytes in response to tissue hypoxia. Similarly, intermediate-phenotype hepatocytes, which may constitute an intermediate stage of dedifferentiation of centrizonal hepatocytes, were reported in conditions demonstrating centrilobular scarring, such as chronic venous outflow obstruction and NASH [34, 38, 49, 50]. Thus, our result is in keeping with a prior study [49] and supports that centrizonal ischemic injury is involved in the fibrogenesis of chronic hepatitis C.

In our NASH control group, no correlation between the *M* score and fibrosis was found, contradicting Gill et al.'s study [34]. In their study, cases with low fibrosis stage (NASH CRN stages 0 and 1a) were excluded, while 13 of 22 (59%) cases were NASH CRN stage 0 or 1a in our study. The predominance of low stage fibrosis and low case number may have contributed to the discrepant result in our study.

We evaluated the utility of central portalization as a histologic marker to identify metabolic risk factors in steatotic HCV biopsies. However, neither *M* nor *D* score identified patients with obesity, type 2 DM, and hyperlipidemia in the study group. Therefore, central portalization cannot be used as a surrogate marker to identify patients with risk factors for NASH in steatotic HCV biopsies. Neither the history of treatment nor HCV genotype 3 correlated with *M* or *D* scores. Likewise, neither the *M* score nor *D* score predicted adverse outcome.

The negative result might be due to insufficient clinical data; the information was inconsistent or unavailable in the electronic medical records in some cases. For example, 6 patients subsequently developed clinical NASH and type 2 DM during the follow-up period, suggesting that the biopsies may have been performed at the subclinical stage of insulin resistance. Or, the negative result may be due to the confounding alcohol history, which may have contributed to centrizonal injury. However, neither *M* score nor *D* score differed between the patients with and without alcohol history, making this possibility unlikely. An alternative and more reasonable explanation is that the mechanism of central zone injury in steatotic chronic hepatitis C is not simply attributable to the risk factors for NASH.

NASH biopsies tend to show accentuated steatosis in zone 3, in contrast to randomly distributed steatosis of HCV biopsies. Hence, zonation of steatosis may be used as a histologic marker to identify HCV patients with concomitant metabolic risk factors. In this study, the zonation of steatosis was evaluated without prior knowledge of the clinical information. However, only 6 cases showed zonation of steatosis in our study group. Moreover, the evaluation of zonation was challenging in cases of advanced fibrosis and marked steatosis, which preclude a standardized approach. This observation suggests that the utility of zonation as a surrogate histologic marker to detect metabolic risk factors is limited.

Our study is not without limitations. Many cases of advanced fibrosis were excluded from the statistical analysis due to low number of recognizable central zones. The inverse correlation between the number of central zones and fibrosis clearly demonstrates the interpretational challenge. Also, any zone with CK19 positivity was excluded from the central zone count. In brief, lobular CK19 positive cells may originate from the adjacent portal tracts by branching and migration of portal cholangiocytes, in conditions of predominant portal injury, such as chronic hepatitis C [38, 51]. Alternatively, the lobular CK19 positive cells may represent dedifferentiating centrizonal hepatocytes, expressing progenitor cell phenotype via CK19 expression, as seen in conditions of predominant centrizonal injury [34, 38]. Regardless, all CK19 positive zones were excluded to minimize the chance of counting portal tracts as central zones, further reducing the number of central zones. Lastly, higher stage fibrosis was overrepresented in the study group compared to the HCV control group, and the case number was low in the HCV control group. These factors may have contributed to the lack of correlation between the *M* score and fibrosis in the HCV control group.

## 5. Conclusions

In summary, our study demonstrates that central zone injury represented by central portalization is positively correlated with fibrosis in steatotic chronic hepatitis C biopsies. The mechanism of central zone injury and fibrogenesis in steatotic chronic hepatitis C does not appear to be solely attributable to the metabolic risk factors for NASH; thus, central portalization cannot be used as a surrogate marker to identify these patients.

## Figures and Tables

**Table 1:** Clinical data, steatosis, and fibrosis of steatotic HCV (>10% steatosis) study group and two control groups—^*^ *P* < 0.05 by Fisher's exact test, statistically significant; the information regarding risk factors was unavailable or incomplete in 9 patients of the study group. ^#^HCV genotype was known in 44 cases in the study group and 17 cases in HCV control group. M: male; F: female; DM: diabetes mellitus; obesity: body mass index (BMI) of ≥30 kg/m^2^; n/a: not applicable; Ishak: Ishak's fibrosis staging system [35]; NASH CRN: NASH Clinical Research Network's fibrosis staging system for NASH [36].

	Study group	Control group	
	HCV with >10% steatosis	HCV with ≤10% steatosis	NASH, no HCV
Number of cases	65	20	22
Age	48	47	43
M : F	49 : 16	17 : 3	13 : 9
Type 2 DM	14 (22%)	1 (5%) (*P* = 0.11)	5 (23%) (*P* = 1.00)
Obesity	33 (51%)	5 (25%) (*P* = 0.07)	20 (91%) (*P* = 0.001^*^)
Alcohol	32 (49%)	8 (40%) (*P* = 0.61)	4 (18%) (*P* = 0.01^*^)

HCV genotype^#^			
1a/1b	34 (77%)	17 (100%) (*P* = 0.049^*^)	n/a
2a/2b	3 (7%)	0 (*P* = 0.55)	n/a
3	7 (16%)	0 (*P* = 0.17)	n/a

Steatosis			
<33%	22 (34%)	20 (100%)	10 (46%) (*P* = 0.44)
33–66%	30 (46%)	0	3 (14%) (*P* = 0.01^*^)
>66%	13 (20%)	0	9 (41%) (*P* = 0.09)

Fibrosis	Ishak	Ishak	NASH CRN
	0–2: 23 (35%)	0–2: 13 (65%) (*P* = 0.04^*^)	0-1: 14 (64%)
	3-4: 23 (35%)	3-4: 6 (30%) (*P* = 0.79)	2: 4 (18%)
	5-6: 19 (29%)	5-6: 1 (5%) (*P* = 0.03^*^)	3: 2 (9%)
			4: 2 (9%)

**Table 2:** Clinical parameters versus fibrosis, steatosis, and nonalcoholic fatty liver disease (NAFLD) activity score [36] in the study group by Student's *t*-test. Present: with the clinical parameter; absent: without the clinical parameter; Ishak: Ishak's fibrosis staging system [35]; ^∗^statistically significant (*P* < 0.05); type 2 DM: type 2 diabetes mellitus.

	Clinical parameter	Present	Absent	*P* value
Fibrosis (Ishak)	Obesity	2.9	3.9	*P* = 0.058
	Type 2 DM	3.3	3.3	*P* = 0.918
	Alcohol	3.6	3.0	*P* = 0.193

Steatosis (%)	Obesity	56%	45%	*P* = 0.030^*^
	Type 2 DM	49%	50%	*P* = 0.880
	HCV genotype 3	50%	48%	*P* = 0.776

NAFLD activity score	Obesity	5.3	4.7	*P* = 0.09
	Type 2 DM	4.9	5.0	*P* = 0.76
	Hyperlipidemia	5.8	4.9	*P* = 0.08
	Alcohol	4.8	5.2	*P* = 0.25

**Table 3:** Pearson's correlation coefficient (*r*) and *P* values for histologic parameters. NASH: nonalcoholic steatohepatitis; NS: not statistically significant (*P* ≥ 0.05); ^*^ *P* < 0.05: statistically significant; NAFLD: nonalcoholic fatty liver disease [36]; *M* score and *D* score: see Section 2.4 for definition.

	Parameters	*r*	*P* value
**Study group** HCV with steatosis >10%	Central zone versus fibrosis	−0.38	0.0018^∗^
	Central zone versus steatosis		NS
	Fibrosis versus steatosis		NS
	Fibrosis versus NAFLD activity score		NS
	*M* score versus fibrosis	0.38	0.008^∗^
	*D* score versus fibrosis	0.36	0.013^∗^
	*M* score versus steatosis		NS
	*D* score versus steatosis		NS
	*M* score versus NAFLD activity score	0.30	0.041^∗^
	*D* score versus NAFLD activity score		NS

**HCV control group** HCV with ≤10% steatosis	*M* score versus fibrosis		NS
	*D* score versus fibrosis	0.54	0.036^∗^
	*M* score versus *D* score	0.49	0.06

**NASH control group** NASH, no HCV	*M* score versus fibrosis		NS
	*M* score versus NAFLD activity score		NS
	*M* score versus steatosis		NS
